# Addressing knowledge gaps and prevention for tuberculosis-infected Indian adults: a vital part of elimination

**DOI:** 10.1186/s12879-018-3116-7

**Published:** 2018-05-02

**Authors:** Andrea DeLuca, Gauri Dhumal, Mandar Paradkar, Nishi Suryavanshi, Vidya Mave, Rewa Kohli, Shri Vijay Bala Yogendra Shivakumar, Vidula Hulyolkar, Archana Gaikwad, Ashwini Nangude, Geeta Pardeshi, Dileep Kadam, Amita Gupta

**Affiliations:** 1 Johns Hopkins Bloomberg School of Public Health, International Health, Baltimore, MD USA; 2 Byramjee-Jeejeebhoy Government Medical College-Johns Hopkins University Clinical Trials Unit, Pune, India; 3 Division of Infectious Diseases, Department of Medicine, Johns Hopkins University School of Medicine, Baltimore, MD USA; 4 Byramjee-Jeejeebhoy Government Medical College, Pune, India; 5 Department of Community Medicine, Vardhman Mahavir Medical College and Safdarjung Hospital, New Delhi, India; 6 Center for Tuberculosis Research, Johns Hopkins University, CRB-2, 1550 Orleans Street, Baltimore, MD 21287 USA

**Keywords:** Tuberculosis prophylaxis, Contacts, Access

## Abstract

**Background:**

India plans to eliminate tuberculosis (TB) by 2025, and has identified screening and prevention as key activities. Household contacts (HHCs) of index TB cases are a high-risk population that would benefit from rapid implementation of these strategies. However, best practices for TB prevention and knowledge gaps among HHCs have not been studied. We evaluated TB knowledge and understanding of prevention among tuberculin skin-test (TST) positive HHCs. While extensive information is available in other high-burden settings regarding TB knowledge gaps, identifying how Indian adult contacts view their transmission risk and prevention options may inform novel screening algorithms and education efforts that will be part of the new elimination plan.

**Methods:**

We approached adult HHC to administer a questionnaire on TB knowledge and understanding of infection. Over 1 year, 100 HHC were enrolled at a tertiary hospital in Pune, India.

**Results:**

The study population was 61% (*n* = 61) female, with a mean age of 36.6 years (range 18–67, SD = 12). Education levels were high, with 78 (78%) having at least a high school education, and 23 (24%) had at least some college education. Four (4%) of our participants were HIV-infected.

General TB knowledge among HHC was low, with a majority of participants believing that you can get TB from sharing dishes (70%) or touching something that has been coughed on (52%). Understanding of infection was also low, with 42% believing that being skin-test positive means you have disease. To assess readiness for preventive therapy, we asked participants whether they are at a higher risk of progressing to active disease because of their LTBI status. Fifty-four (55%) felt that they are at higher risk. Only 8% had heard of preventive therapy.

**Conclusion:**

Our TB knowledge survey among HHCs with evidence of recent exposure found that knowledge is poor and families are confused about transmission in the household. It is imperative that the Indian program develop tools and incentives that can be used to educate TB cases and their families on what infected HHCs can do to prevent disease, including preventive therapy.

## Background

India plans to eliminate tuberculosis (TB) by 2025 [1]. The National Strategic Plan for Elimination identifies treatment of latent TB infection (LTBI) as a component, stating, ‘Treating 40 per cent of the population for LTBI is neither rational nor practicable, thus emphasizing the need for a focused approach’ [2]. As part of their Prevent strategy, the plan recommends treatment for LTBI specifically for contacts of bacteriologically-confirmed cases. Targeting household-members is a decades-old strategy with proven effectiveness as a way to interrupt transmission, reduce prevalence and ‘get to zero’ [3–7]. With over two million cases of TB per year [8], India faces a substantial challenge in reaching those most at-risk of developing active disease and providing preventive therapy [9, 10].

Most experts agree that the global information on prophylaxis is exhaustive – it saves lives [11–15]. India does not provide statistics on provision of isoniazid preventive therapy (IPT) in the government sector [8]. The in-country evidence base on preventive therapy is limited to assessments of implementation [16–18], one treatment trial that compared options for HIV+ adults [19], and a study from the 1970s that attempted a Comstock-type-model of mass preventive therapy in villages outside of Bangalore [20]. There are no published data or national statistics that directly address treatment of LTBI among adult household contacts of TB cases in India. For the other known risk groups, several studies have demonstrated low adherence to national guidelines for pediatric contacts and HIV+ adults [21–23]. Best practices for IPT and knowledge gaps among high-risk household contacts has not been studied. While extensive information is available in other high-burden settings regarding TB knowledge gaps, identifying how Indian adult contacts view their transmission risk and prevention options may inform novel screening algorithms and education efforts that will be part of the new elimination plan. We surveyed household contacts of pulmonary TB cases in India with a focus on understanding the perceptions of TB infection and prevention among skin-test positive individuals.

## Methods

We approached 100 sequentially enrolled skin-test positive adult household contacts (HHC) of newly diagnosed adult pulmonary TB (PTB) patients who lived in the same household at least 3 months prior to index TB diagnosis in a cohort study at the Byramjee Jeejeebhoy Government Medical College (BJGMC), in Pune, India for participation in a sub-study. More than one person could be enrolled per household. CTRIUMPH (Cohort for TB Research with Indo-US Medical Partnership), an NIH and Indian Government funded study, enrols newly diagnosed adult PTB cases seen at a government clinic and their HHCs to be followed over a 24-month period [24]. As part of enrolment, all consenting HHCs are screened for LTBI using a tuberculin skin test (TST) and a Quantiferon Gold-in-Tube (QGIT) blood test manufactured by Qiagen. Because QGIT can only be used in India for research purposes [25], we approached tuberculin skin-test positive HHCs, with an induration of 5 mm or more. We used a verbal consent process in the local language for agreement to participate in a sub-study that involved administration of a questionnaire, and none of those approached refused enrolment. The Johns Hopkins Medicine Institutional Review Board (IRB) and the BJGMC Clinical Trials Unit IRB approved a verbal consent process, as the study involved minimal risk. Between December 2015–March 2017, a trained social scientist interview-administered the questionnaire using a validated TB knowledge scale developed by the Centers for Disease Control [26], followed by questions adapted from Butcher and colleagues [27] that included space for open-ended responses. The authors used the Butcher and colleagues questionnaire as described in *BMJ Research Notes* because it was the only publically available questionnaire specifically focused on latent TB infection with insights for perception and knowledge. The original questionnaire is generalizable to patients with LTBI. Not all respondents provided an open-ended response following quantitative questions, and not every respondent answered each question, so responses and percentages are noted for each result. We analyzed TB knowledge responses using Stata 14.0 (StataCorp, College Station, TX) using chi2 to test for statistically significant differences between sub-populations of interest (HIV infected, gender, etc.), and identified quotes that support the key findings. Basic demographics, including age, sex, education level, as well as documented HIV-status, were abstracted from the CTRIUMPH database. The sub-study data is available from the corresponding author upon request.

### Ethics, consent, and permissions

This research was approved by BJGMC Clinical Trials Unit ethics review committee and the Johns Hopkins IRB. All participants were verbally consented and agreed to participate.

## Results

The study population was 61% (*n* = 61) female, with a mean age of 36.6 years (range 18–67, SD = 12). Education levels were high, with 78 (78%) having at least a high school education, and 23 (24%) had at least some college education. Eleven participants were illiterate (*n* = 12%). Ten individuals (11%) were unemployed at the time of their interviews, and 19 (20%) were housewives. Four (4%) of our participants were HIV-infected. Seventy-eight households were individually represented, with 22 households having more than one member enrolled in the sub-study.

Responses to the TB knowledge questions (Table 1) indicate that our participants understood that crowding puts them at risk for developing TB (80%), a person with TB can look and feel fine (68%), and that taking medication can cure almost all forms of TB (89%).

**Table 1:** Stratification of TB knowledge by sub-groups of interest among Household Contacts

TB Knowledge Question	Response^a^	Gender N (%)			HIV Status N (%)			At Least a High School Education N (%)			Total N (%)
		Male	Female	χ^2^; *p* value	+	_	χ^2^; p value	Yes	No	χ^2^; p value	
It is easier to get TB by living in crowded conditions	True	30 (83)	46 (79)	1.92; 0.38	2 (50)	71 (80)	2.87; 0.24	64 (83)	15 (68)	4.87; 0.09	79 (80)
	False	4 (11)	11 (19)		2 (50)	15 (17)		10 (13)	7 (32)		17 (17)
	Uncertain	2 (6)	1 (2)		0	3 (3)		3 (4)	0		3 (3)
A person with TB disease can look and feel fine	True	24 (65)	38 (66)	4.87; 0.08	2 (50)	60 (67)	1.20; 0.55	50 (64)	17 (77)	1.46; 0.48	68 (68)
	False	8 (22)	16 (28)		1 (25)	22 (24)		20 (26)	4 (18)		23 (23)
	Uncertain	5 (14)	4 (7)		1 (25)	8 (9)		8 (10)	1 (5)		9 (9)
A positive TB skin test means that you already have disease	True	23 (40)	17 (46)	0.54; 0.76	3 (75)	37 (41)	2.07; 0.36	32 (41)	10 (46)	1.33; 0.51	42 (42)
	False	23 (40)	12 (32)		1 (25)	33 (37)		31 (40)	6 (27)		37 (37)
	Uncertain	12 (21)	8 (22)		0	20 (22)		15 (19)	6 (27)		21 (21)
A person can get TB by sharing dishes or utensils with someone who has TB	True	28 (76)	38 (66)	1.12; 0.57	4 (100)	61 (68)	1.86; 0.39	55 (70)	15 (68)	1.86; 0.39	70 (70)
	False	7 (19)	15 (26)		0	22 (24)		20 (26)	3 (14)		23 (23)
	Uncertain	2 (5)	5 (9)		0	7 (8)		3 (4)	4 (18)		7 (7)
I am afraid of infecting others	True	20 (54)	23 (40)	2.39; 0.30	1 (25)	42 (47)	2.13; 0.35	32 (41)	12 (55)	1.39; 0.50	46 (46)
	False	15 (41)	28 (48)		2 (50)	42 (47)		29 (50)	8 (36)		45 (45)
	Uncertain	2 (5)	7 (12)		1 (25)	6 (6)		7 (9)	2 (9)		9 (9)
Health care providers should be giving me medication for LTBI	True	28 (76)	41 (71)	0.28; 0.86	4 (100)	66 (73)	1.43; 0.70	59 (77)	14 (64)	4.92; 0.18	73 (73)
	False	8 (21)	15 (26)		0	22 (24)		18 (23)	6 (27)		24 (24)
	Uncertain	1 (3)	2 (3)		0	2 (3)		1 (2)	1 (4)		2 (2)

^a^Not every participant had demographic information available, so denominators provided for each **χ**^**2**^ calculation

A majority responded incorrectly to the TB transmission questions of getting TB by sharing dishes or utensils with someone who has TB (70%) or by touching something that someone with TB has coughed on (52%). When asked if they were afraid whether they may infect others (as a person with LTBI), 46 (46%) said yes. One illiterate 48-year old female was not worried, saying, “*No, I will take care of it. This is our family disease since last 15 years I am taking care of TB patients. Firstly, my husband had this TB disease, then my son became ill due to TB since last 3 years and he is not yet cured. Therefore my daughter got affected as we are living in one household.”*

We asked the participants whether they understood what is the cause of TB, 70 (71%) either did not know or were unsure of its cause. Open-ended responses to this question highlighted the various beliefs about its causation. Ten respondents mentioned cough, seven mentioned living with or coming in contact with a TB patient, and five drug or alcohol abuse. A 31-year-old woman with a 9th standard education remarked, *“Yes, TB may be caused due to fever, cough, vomiting of blood, due to air, if we came in contact outside with others.*” Another 26 year-old youth with a college education said, *“Yes. TB can be caused due to dust, due to pollution. TB may spread due to the coughing of the diseased TB person”*’. When asked if they understood the difference between TB infection and disease, (90%) did not or were unsure of the difference. A 19-year old college-going woman responded, “*Disease means which is already present in the body. Infection means it is caused due to other person’s infection”.* Another respondent, an illiterate 48-year old woman, said, “*Yes, disease means to have cough and sneezing, whereas infection means spread of disease”*. A 48-year-old man with a 9th standard education said, *“Yes, infection is a first stage and disease is serious condition. We have to take treatment in both conditions.”*

Forty-three (43%) thought that they have the germ that causes TB in their bodies, and the remaining 57% were unsure or did not know if they had the germ that causes disease in their bodies. One 27 year-old male with a primary level education said, *“Yes, Now [my] wife is infected by TB. So [I]might get affected. [We] are living in one household. [I have] a fear of coughing.”*

In response to a question on whether a positive tuberculin skin test means that you already have TB disease, 42% believed it be to be true while 21% did not know, and 38% thought it is not true. We also asked whether being infected with TB does not mean that you are sick, and 52 (52%) said true.

To assess readiness for preventive therapy, we asked participants whether they are at a higher risk of progressing to active disease because of their LTBI status. Fifty-four (55%) felt that they are at higher risk. One 27-year primary educated man said, *“No, I don’t think I will get affected. I am living in other room and wife is in another room.”* A 45-year old woman with a 10th standard education said, *“Yes, because bacteria is present in our body. So when we will develop symptoms like weight loss, loss of appetite, fever then we may get disease.”* A 24-year old female with a 9th standard education said, *“Yes, could not understand anything. I feel that I might have disease*.” When we stratified by HIV status, all HIV-infected individuals (100%) responded that they were at a higher risk for disease progression. Ninety-two percent of our participants have never heard of preventive therapy. Among the eight who reported hearing about it, one 31-year old woman with a 9th standard education said, “*They had suggested medicine for children but our reports are normal so we did not opt for it.”* Another 42-year old illiterate female said, “*Doctors started the treatment but the medicines are not there…we don’t have any problem, we are okay.”*

We asked if health care providers should be giving medication for their LTBI status. Seventy-three (73%) of respondents said yes, and again all four of the HIV+ respondents said yes. A 33-year old man with a 9th standard education said, *“Yes, if body has bacteria, and will get treatment early then it may prevent the future consequences”.* Another 34-year old man with a 12th standard education said, “*Yes, to become healthy, even if we do not have disease now but it may cause in future.”* A 31-year old female with a 10th standard education said, *“Yes, I had asked about any tablets to doctors but they said it is not required, you can take calcium, protein rich food….I have informed doctors that I am ready to take any medication.”*

Lastly, we assessed if TB knowledge questions were associated with age, education, sex, and HIV status, using the chi-squared test, and did not find any significant differences between those sub-groups (Table 1).

## Discussion

Our TB knowledge survey among a sample of 100 Indian HHCs with evidence of LTBI and recent exposure to someone in their household with active PTB, had several important findings. First, TB knowledge around transmission was poor, with less than 30% knowing that TB cannot be transmitted by sharing food or utensils. Our data suggest that it is urgent to discuss how TB spreads in the household and what can be done to prevent transmission. Individual responses indicated a high level of confusion/misinformation about how HHC can get TB, and there are issues of stigma that have been prevalent in other hot spots around the globe [28, 29]. Generalized stigma in India is well-know and may lead to rejection and social isolation [30]. With 46% of our population afraid of infecting others, including some of them not sleeping in the same room with a spouse, the program would do well to incorporate knowledge-shaping and attitude-changing interventions that have been proven effective, including mass media messaging and support groups [31].

The Revised National Tuberculosis Control Program (RNTCP) will need to find effective ways to connect the link between disease transmission and infection as a foundation for starting preventive therapy for TB. One of the previous studies completed in India demonstrated that poor TB knowledge was a barrier to IPT [21]. Training and education interventions have been assessed in other countries with a high TB burden has shown that improved TB knowledge can change the way high risk groups and health care providers approach TB prevention, and can be considered for future research in India [32–35].

Secondly, there is confusion about what LTBI is and what a positive tuberculin skin test means. Despite some of these knowledge gaps, most were interested and willing to take preventive therapy. Yet the vast majority stated that they were not aware of preventive therapy and had not been informed about it. If 57% of our population didn’t understand infection status despite having positive tuberculin skin-test, there is much work to be done with counsellors and health care providers. We recommend that the RNTCP screen all HHCs of adult pulmonary TB cases as a high-priority risk group and provide education on TB transmission while doing it. As recently infected LTBI poses at higher risk for TB disease progression in 12–18 months from the time of LTBI acquisition, it makes it a high priority to screen HHCs of TB patients who are constantly exposed to TB bacilli in their household. Because the science of progression from infection to disease is rapidly changing, it may be best to screen these HHC’s to rule out TB disease and focus on prevention strategies and communicate the importance of “treating TB infections” in absence of TB symptoms.

Most importantly, our data suggest that tuberculin skin test positive HHCs are ready to take prophylaxis. If the RNTCP is able to systematically screen HHCS of bacteriologically confirmed cases and offer preventive therapy (e.g. 6 to 9 months of isoniazid/3 months of weekly INH + rifapentine (3HP)/4 months of daily rifampin), as per the National Strategic Plan for TB Elimination, our data give reason to believe that uptake would be good. Even in the small group of participants who knew of preventive therapy, issues of isoniazid stock-outs and poor education about why taking IPT is important were noted. If India has the infrastructure to support the treatment of millions of active TB cases, simple, effective mechanisms for screening and offering preventive therapy to HHCs can also be incorporated in to the program. Removing the requirement for a tuberculin skin test would overcome known barriers, and if the RNTCP keeps it as a requirement, recent studies suggest that the interpretation can be done at home [36]. With 73% of our population willing to take preventive therapy in the absence of good understanding of TB transmission and infection, there is reason to believe that evidence-based campaigns will further improve willingness to take prophylaxis and adherence once treatment for TB prophylaxis is initiated [37].

This study has several limitations, most notably our participants were a part of a larger cohort study who had in-depth assessments with health care providers. These findings may not be generalizable to HHCs who have never had an interaction with the health care system. We also had a limited sample size, and are not able to determine any associations between key TB knowledge and demographic characteristics.

## Conclusion

Our study shows that high-risk HHCs have a poor understanding of how TB transmission occurs, and a majority are unsure of what their positive skin test means. Most of the HHCs with an index TB case in their household are willing to take preventive therapy. To support the Indian government’s plan for eliminating TB, we recommend routine screening for TB and mechanisms for offering preventive to HHCs of active PTB cases. In a country where more than 1000 people die every day from TB, the urgency for action is real and must be met by the government and private sector with commitment and resources. Mandatory program indicators for HHC screening, GeneXpert point-of-care testing, and education for and delivery of preventive therapy are proven mechanisms that can be implemented now. High-risk contacts cannot afford to wait.

## Abbreviations

3HP: 3 months of isoniazid and rifapentine
BJGMC: Byramjee jeejeebhoy government medical college
CTRIUMPH: Cohort for TB research with indo-US medical partnership
HHC: Household contacts
HIV: Human immunodeficiency virus
IPT: Isoniazid preventive therapy
IRB: Institutional review board
LTBI: Latent tuberculosis infection
PTB: Pulmonary tuberculosis
QGIT: Quantiferon gold-in-tube
RNTCP: Revised national tuberculosis control program
